# Musculoskeletal Occupational Injuries in Orthopaedic Surgeons and Residents

**DOI:** 10.5704/MOJ.2003.004

**Published:** 2020-03

**Authors:** KSK Tan, EBK Kwek

**Affiliations:** 1 Department of Orthopaedic Surgery, Tan Tock Seng Hospital, Singapore; 2 Department of Orthopaedic Surgery, Woodlands Health Campus, Singapore

**Keywords:** occupational, injury, musculoskeletal

## Abstract

**Introduction:**

Orthopaedic surgeons are significantly predisposed to musculoskeletal injury, and these injuries can have negative effects on surgeon function and patient outcomes. While this phenomenon has been studied in the non-Asian population of surgeons, no study has been carried out in the local or regional Asian setting. The aim of this study was to determine the prevalence, characteristics and associations of occupational injuries in orthopaedic surgeons and residents, and to assess its functional impact.

**Materials and Methods:**

The Nordic Musculoskeletal Questionnaire was sent out to all orthopaedic consultants and residents at two institutions in Singapore, via an email link to an online survey. Separately, further questions on symptom description, severity and treatment were surveyed. Additional information like age, gender, height and weight were obtained as well.

**Results:**

A total of 87.5% respondents have at least one injury. Neck symptoms (66.1%) were the most prevalent, and back symptoms had the highest median severity score (4.5/10). The 74.1% of these injuries were reported as directly attributable to work. Age was found to be associated with an increase in the total number of anatomical areas affected (p = 0.016). A seated operating position was associated with more severe back pain (p = 0.040).

**Conclusion:**

There is a high prevalence of occupational injuries sustained in our population of orthopaedic surgeons. Neck symptoms, followed by back and wrist symptoms, were the predominant symptoms in our population. Targeted ergonomic interventions may be considered to prevent specific musculoskeletal injuries in our population of orthopaedic surgeons.

## Introduction

The practice of orthopaedics has been associated with many occupational hazards, such as radiation exposure, chemicals, surgical smoke, noise and physical injury^1^. Of these occupational risks, orthopaedic surgeons are considerably predisposed to musculoskeletal injury^2^, and the ergonomics of surgical practice contributes significantly to this. In the operating room, the work postures adopted by surgeons introduce significant ergonomic stress load, predisposing them to injury^3, 4^. Other factors such as prolonged standing, repetitive actions^5^ and the use of surgical instruments^6^ are also known ergonomic causes of injury. As compared to general surgeons, orthopaedic surgeons were found to have a higher prevalence of musculoskeletal injuries^7^. Studies done in specific groups of orthopaedic subspecialties^8-11^ and even orthopaedic residents^12^ also reported high levels of musculoskeletal symptoms.

As of yet, no study has been carried out on orthopaedic surgeons in the Asian population. Ethnic differences in body structure may account for differences in the prevalence and severity of musculoskeletal symptoms^13^. Hence the aim of our study is to determine the prevalence and characteristics of these musculoskeletal injuries in our orthopaedic surgeons and residents, and to assess their level of impact on surgeon function, in comparison to the previous studies done in non-Asian populations.

## Materials and Methods

Our study population consisted of all 80 orthopaedic residents and consultants within two tertiary hospitals in Singapore. All respondents received an email invitation to complete an anonymous online survey, and received periodic email reminders to request for their participation.

The Nordic Musculoskeletal Questionnaire^14^ was used to assess the symptomatology of each subject. The questionnaire consists of a set of questions assessing for musculoskeletal complaints in nine anatomical areas of the body, and has been widely used and validated in multiple studies^15^. Separate questions were included about symptom description, symptom severity, symptom treatment and the respondent’s subjective judgment if the symptom was directly attributed to work. Respondents were also asked if they felt their positive symptoms have threatened their career or chosen subspecialty, or if they have prevented them from working optimally. Respondent age, gender, height and weight, weekly operating hours, as well as practice factors (hand dominance, operating position, use of eyewear) were surveyed as well.

The study was approved by the Institutional Review Board before the commencement of the survey. The survey was administered via an email link to an online survey website. Data was collected over an eight-week period. Statistical analysis was performed using STATA version 11.0 software [Stata Corporation, College Station, TX].

## Results

Fifty-six (70.0%) of the pool of 80 orthopaedic surgeons and residents completed the survey. Thirty-four (60.7%) of the respondents were residents, 20 (35.7%) were consultants and 2 (3.6%) did not declare their training status. Of the 20 consultants, all subspecialties were represented with the exception of paediatric orthopaedics and musculoskeletal oncology. The median age of the respondents was 33 years. Thirty-six (64.3%) of the respondents were aged between 28-35, with 14 (25.0%) aged between 35-50 and 6 (10.7%) between 50-70. Fifty-one (91.1%) of the respondents were male.

The median weekly operating hours were 16.0 hours a week. Fifty-four (96.4%) of the respondents were right-handed, and 12 (21.4%) of the respondents used either lead glasses or magnification while operating. Forty-eight (85.7%) of them operated in a standing position.

**Fig. 1:**
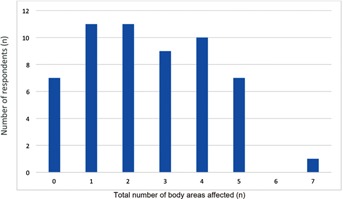
Number of respondents with total number of body areas affected.

**Fig. 2:**
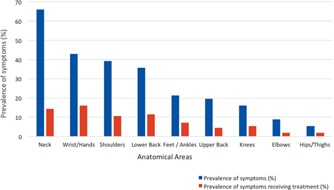
Prevalence of symptoms for each anatomical area.

Forty-nine (87.5%) of all the respondents were symptomatic, of which 38 (67.9%) had 2 or more anatomical areas affected (Fig. 1). Amongst the 9 anatomical areas assessed for symptoms, neck symptoms were the most prevalent, with 37 (66.1%) of respondents complaining of neck pain. This was followed by hands/wrist symptoms (42.9%) and shoulder symptoms (39.3%) (Fig. 2).

When looking at the treatment sought for each symptom, wrist and hand symptoms received the most treatments, followed by neck, lower back, shoulders (Fig. 2). For all the treated symptoms, physiotherapy was the most prevalent treatment used (57.1%).

When comparing the severity of these symptoms, lower back symptoms had the highest median severity of 4.5 out of a score of 10. This was followed by upper back symptoms with a median severity score of 4.0.

We noted that that 74.1% of all symptoms were reported as directly attributable to work, and of these symptoms, 33.3% of the respondents felt that their symptoms prevented optimal function, and 39.2% of the respondents felt that their symptoms will have lasting effects on their career.

Age was found to be associated with an increase in the total number of anatomical areas affected (p = 0.016), but otherwise was not correlated with the severity of symptoms. There were no significant associations found between gender, height, weight and BMI with the number and severity of symptoms.

Years of practice, subspecialty, training status, and operating hours per week were also not found to be significant factors in influencing symptoms. Of note, a seated operating position was associated with an increased severity of lower back pain (p = 0.040).

## Discussion

This study has shown that there is a high prevalence of musculoskeletal symptoms in our population of orthopaedic surgeons, with predominantly neck and upper limb symptoms.

Age has been shown to be associated with an increase in the number of anatomical areas affected, which is likely contributed by age-related physical changes which increases the predisposition to injury^16^.

In comparison to the previous studies, our population has a significantly higher prevalence of musculoskeletal symptoms (87.5%). Davis *et al* reported that 44.0% of their subjects sustained one or more musculoskeletal injuries in their American population of surgeons and Alqahtani *et al* found that 66.0% of arthroplasty surgeons and 67.0% of trauma surgeons in their population of Canadian and Saudi surgeons had musculoskeletal injuries^2, 9^.

Neck pain was noted to be the most reported symptom in our study, followed by wrist and shoulder symptoms. This is similar to the findings by Davis *et al* who noted neck pain (59.4%) as the most frequent symptom, followed by lower back pain (54.8%) and shoulder pain (34.4%)^2^. However, Alqahtani *et al* noted lower back pain (27.9%) as the most frequent symptom, followed by elbow and shoulder pain (14.2%)^9^.

The cause of the higher prevalence of symptoms as well as the difference in the distribution of symptoms in our population of orthopaedic surgeons as compared to the other studies are likely to be multifactorial, but it might be suggested that the ethnic differences in body structure in our population as compared to the non-Asian population increases the overall risk for musculoskeletal injury as well as the risk for specific injuries of the neck and hands/wrist. The study also noted that a seated posture is associated with worse back pain. Targeted ergonomic interventions with due considerations to seating posture and duration, viewing angles of the surgical field, and hand/grip positioning of tools may be effective in our population of orthopaedic surgeons.

Although the increased prevalence of symptoms in our population of surgeons may be alarming, a large proportion of our surgeons (66.7%) are still able to cope and function with their symptoms. This is comparable to the previous studies where Davis *et al* reported that 63.9% of their respondents felt that their symptoms had no impact on operating room performance, and Alqahtani *et al* reported that 73.3% of their respondents did not require time off work due to their symptoms^2, 9^. The tolerance of symptoms could be attributed to the median severity of symptoms being at a manageable level (the highest median severity symptom score was 4.5/10 for lower back pain).

Given the significant prevalence of musculoskeletal symptoms in our population of orthopaedic surgeons compared to other populations, there should be a stronger impetus to deliberately implement measures to improve ergonomics inside the operating room^12^. There are current recommendations to utilise ergonomic body support, and to increase the usage of power tools over manual tools^1, 17^. The design of surgical instruments can also be improved to increase the degrees of freedom, which contributes to better dexterity^18^.

Ergonomic education programmes have also been proposed to be incorporated into residency programs as well^12^. Preventive measures can also be implemented outside of the operating environment, like advocating a lifestyle of regular exercise, which has been shown to confer a protective effect from work-related injuries^19, 20^.

Our study has several limitations. The use of a self-reported measure introduces bias into the results, as the respondents may choose to under-report or over-report symptoms. Also, it has been noted that prior musculoskeletal symptoms are poorly remembered after some years, and that recall of past symptoms are strongly influenced by current symptoms^21^. The response rate of our study was also less than 80%, with a relatively small sample size that is skewed towards the younger age categories, which may not fully represent our local and regional population of surgeons. Certain confounders were also not factored into our study, such as non-surgical work (clinics, administrative work) which may also contribute towards musculoskeletal symptoms. There is a role for further research in our local population of surgeons to better understand this phenomenon well as to improve ergonomic factors to reduce the risk of these injuries.

## Conclusion

This study shows that there is a high prevalence of musculoskeletal occupational injuries sustained in our population of orthopaedic surgeons as compared to the Western studies. A large proportion of our surgeons are still able to cope and function despite the higher prevalence of symptoms. Ethnic differences in build and body structure between Asians and non-Asians may account for differences in the distribution and severity of symptoms. Targeted ergonomic interventions may be considered to prevent specific musculoskeletal injuries in our population of orthopaedic surgeons.

## References

[1] Lester JD, Hsu S, Ahmad CS (2012). Occupational hazards facing orthopaedic surgeons.. Am J Orthop (Belle Mead NJ)..

[2] Davis WT, Sathiyakumar V, Jahangir AA, Obremskey WT, Sethi MK (2013). Occupational injury among orthopaedic surgeons.. J Bone Joint Surg Am..

[3] Kant IJ, de Jong LC, van Rijssen-Moll M, Borm PJ (1992). A survey of static and dynamic work postures in operating room staff.. Int Arch Occup Environ Health..

[4] Janki S, Mulder EEAP, IJzermans JNM, Tran TCK (2017). Ergonomics in the operating room.. Surg Endosc..

[5] Lin DW, Bush RW, Earle DB, Seymour NE (2007). Performance and ergonomic characteristics of expert surgeons using a face-mounted display during virtual reality-simulated laparoscopic surgery: an electromyographically based study.. Surg Endosc..

[6] Catanzarite T, Tan-Kim J, Whitcomb EL, Menefee S (2018). Ergonomics in Surgery: A Review.. Female Pelvic Med Reconstr Surg..

[7] Mirbod SM, Yoshida H, Miyamoto K, Miyashita K, Inaba R, Iwata H (1995). Subjective complaints in orthopedists and general surgeons.. Int Arch Occup Environ Health..

[8] Auerbach JD, Weidner ZD, Milby AH, Diab M, Lonner BS (2011). Musculoskeletal disorders among spine surgeons: Results of a survey of the Scoliosis Research Society membership.. Spine (Phila Pa 1976)..

[9] Alqahtani SM, Alzahrani MM, Harvey EJ (2016). Prevalence of musculoskeletal disorders among orthopaedic trauma surgeons: An OTA survey.. Can J Surg..

[10] Alqahtani SM, Alzahari MM, Tanzer M (2016). Adult Reconstructive Surgery: A High-Risk Profession for Work-Related Injuries.. J Arthroplasty..

[11] Alsiddiky AM, Alatassi R, Altamimi SM, Alqarni MM, Alfayez SM (2017). Occupational injuries among pediatric orthopaedic surgeons: How serious is the problem?.. Medicine (Baltimore)..

[12] Knudsen ML, Ludewig PM, Braman JP (2014). Musculoskeletal pain in resident orthopaedic surgeons: results of a novel survey.. Iowa Orthop J..

[13] Quintana RM (2017). Work-Related Musculoskeletal Disorders and the Relationship to Ethnicity. Occupational Health [Internet].

[14] Kuorinka I, Jonsson B, Kilbom A, Vinterberg H, Biering-Sorensen F, Andersson G (1987). Standardised Nordic questionnaires for the analysis of musculoskeletal symptoms.. Appl Ergon..

[15] Descatha A, Roquelaure Y, Chastang JF, Evanoff BA, Melchior M, Mariot C (2007). Validity of Nordic-style questionnaires in the surveillance of upper-limb work-related musculoskeletal disorders.. Scand J Work Environ Health..

[16] Lee DJ, Fleming LE, Leblanc WG, Arheart KL, Ferraro KF, Pitt-Catsouphes M (2012). Health status and risk indicator trends of the aging US health care workforce.. J Occup Environ Med..

[17] Albayrak A, van Veelen MA, Prins JF, Snijders CJ, de Ridder H, Kazemier G. (2007). A newly designed ergonomic body support for surgeons.. Surg Endosc..

[18] Pandve HT (2014). Role of Ergonomics In Health Care.. J Ergonomics..

[19] Lagerström M, Wenemark M, Hagberg M, Hjelm EW (1995). Occupational and individual factors related to musculoskeletal symptoms in five body regions among Swedish nursing personnel. Int Arch Occup Environ Health..

[20] van den Heuvel SG, Heinrich J, Jans MP, van der Beek AJ, Bongers PM (2005). The effect of physical activity in leisure time on neck and upper limb symptoms.. Prev Med..

[21] Miranda H, Gold JE, Gore R, Punnett L (2006). Recall of prior musculoskeletal pain.. Scand J Work Environ Health..

